# The epidemiology of myasthenia gravis

**DOI:** 10.25122/jml-2020-0145

**Published:** 2021

**Authors:** Ana-Maria Bubuioc, Aigerim Kudebayeva, Saule Turuspekova, Vitalie Lisnic, Maurizio Angelo Leone

**Affiliations:** 1. Department of Neurology, Nicolae Testemitanu State University of Medicine and Pharmacy Chisinau, the Republic of Moldova; 2. Department of Neurology, Kazakh Medical University of Continuing Education, Almaty, Kazakhstan; 3. Department of Nervous Diseases with course of Neurosurgery, Asfendiyarov Kazakh National Medical University, Almaty, Kazakhstan; 4. Neurology Unit, Fondazione IRCCS Casa Sollievo della Sofferenza, San Giovanni Rotondo, Italy

**Keywords:** myasthenia gravis, epidemiology, autoimmunity, incidence, prevalence

## Abstract

Neuromuscular junction (NMJ) disorders include several dysfunctions that ultimately lead to muscle weakness. Myasthenia gravis (MG) is the most prevalent NMJ disorder with a highly polymorphic clinical presentation and many different faces. Being an autoimmune disease, MG correlates with the presence of detectable antibodies directed against the acetylcholine receptor, muscle-specific kinase, lipoprotein-related protein 4, agrin, titin, and ryanodine in the postsynaptic membrane at the NMJ. MG has become a prototype serving to understand both autoimmunity and the function of the NMJ better. The aim of this review is to synthesize some of the epidemiological data available. Epidemiological data regarding MG are important for postulating hypotheses regarding its etiology and facilitating the description of MG subtypes. Thus, adequate documentation through broad databases is essential. The incidence and prevalence of MG reported around the globe have been rising steadily and consistently over the past decades. Ethnic aspects, gender-related differences, and environmental risk factors have been described, implying that these might contribute to a specific phenotype, further suggesting that MG may be considered an umbrella term that covers several clinical entities.

## Introduction

Neuromuscular junction (NMJ) disorders comprise several dysfunctions that ultimately lead to muscle weakness. Some of these diseases, such as congenital myasthenic syndromes, are genetic. Toxins like botulinum toxin and curare also disrupt the activity at the level of the NMJ. Other NMJ disorders are acquired autoimmune forms, such as myasthenia gravis (MG), Lambert-Eaton myasthenic syndrome (LEMS), and neuromyotonia.

MG is the most frequent NMJ disorder with a highly polymorphic clinical presentation manifesting predominantly, as the etymology suggests (“*mus*” meaning “muscle”, “*asthenia*” meaning “weakness”), with muscle weakness and fatigue [1]. MG represents the largest group of NMJ disorders and occurs as a result of the impairment of neuromuscular transmission [2]. Given its pathogenetic mechanism and specific anatomical location, MG has become both a prototype for autoimmune disorders and a model for understanding the synaptic function [3]. It is an autoimmune B-cell mediated disease and correlates with the presence of antibodies directed against the acetylcholine receptor (AChR), muscle-specific kinase (MuSK), lipoprotein-related protein 4 (LRP4), agrin, titin, and ryanodine in the postsynaptic membrane at the NMJ [3]. MG patients may be categorized into subtypes in order to facilitate the therapeutic process and to estimate the prognosis. Subtypes based on the clinical presentation and seropositivity include early-onset MG, late-onset MG, thymoma, MuSK, LRP4, seronegative, and ocular MG [4].

MG is a rare disease. It is estimated that most neurologists might encounter a patient with MG once in 3–4 years and that their practice would not include more than four patients with MG simultaneously [5]. As a result of this paucity of cases and because of the uneven distribution and difficult data collection, it may be problematic to describe clear epidemiological patterns for rare diseases with multifactorial causes such as MG. Nevertheless, certain tendencies have been observed over the last decades. The number of MG patients is growing, and it has more than doubled in the last 20 years. This rise is mostly due to a greater MG incidence in the elderly, probably because of the better diagnosis, treatment, and increasing longevity of the population. Environmental and genetic causes have also been explored.

Certain epidemiological features of MG are consistent in most studies but have not been explained yet. One of them is the bimodal age distribution with two peaks of incidence: early-onset MG in the third decade (mostly females) and late-onset MG in the elderly (mostly males) [6]. Another epidemiological riddle is the ethnic discrepancy observed when comparing cohorts with different racial origins. The multitude of clinical presentations, as well as the diverse epidemiological aspects, have led clinicians to believe that MG is most likely the result of different diseases that display one indistinguishable clinical picture. The aim of this review is to synthesize some of the epidemiological data available.

### Incidence and prevalence

Based on 35 studies up to 2007 [1], the incidence rate of MG varied from 1.7 to 21.3, with a global rate of 5.3 per million person-years. The pooled incidence rate after 1976 is approximately twice greater than the one before 1976, which is 6.5 vs. 3.5, respectively [7]. In Table 1, we updated this review to 2019, adding 29 studies with a range of 0.15 to 61.33 per million person-years. The global incidence rate of acetylcholine receptor antibody-positive MG ranges between 4 and 18 per million person-years [8]. The incidence of MuSK MG is estimated at 0.1 per million person-years in Holland and 0.32 per million person-years in Greece [1].

**Table 1.** Incidence and prevalence of myasthenia gravis worldwide.

	**Country**	**Study population size**	**Period**	**Number of cases**	**Diagnostic criteria**	**Case ascertainment**	**Incidence per million person-years) Crude rate [95% CIs]**	**Prevalence (per 100 000) Crude rate [95% CIs]**	**Reference**
1.	Argentina	2.890.151, Buenos Aires	2006–2012	60	Clinical assessment, pharmacological tests, electrophysiological studies, anti AChR, anti-MuSK antibodies.	Neuromuscular Diseases section, the HMO electronic medical records at Hospital Italiano de Buenos Aires.	61.33 (95% CI 47.62–79.99)	36.71	[9]
2.	Australia	Nationwide	2009	545	Patients receiving Pyridostigmine during 2009.	The Pharmaceutical Benefits Scheme (PBS) of Australia. The Repatriation Pharmaceutical Benefits Scheme.	24.9	11.7	[10]
3.	Austria	Nationwide	2009	669 - inpatients; 100- outpatients;	Discharge diagnoses.	Austrian hospital discharge register (HDR).	-	- 8.0 inpatient prevalence. -15.69 (95 % CI 13.16–19.42) population prevalence	[11]
4.	Belarus	Nationwide	2012	54	Clinical assessment, pharmacological tests, electrophysiological studies, CT/MRI of the mediastinum, anti-AChR antibodies.	Register of patients with MG.	5.42 (95% CI 4.8–6.04)	10.7	[12]
5.	Canada	13.500.000, Ontario	1996–2013	6750	Hospital discharge with diagnosis of MG or 5 outpatient MG visits and 1 relevant diagnostic test, within 1 year, or 3 pyridostigmine prescriptions, within 1 year.	The Canadian Institute for Health Information (CIHI) discharge abstracts database. The Ontario Health Insurance Program (OHIP) database. The Ontario Drug Benefit (ODB) database. The Ontario Registered Persons Database.	1996 - 27 (95% CI 23–30) 2013 - 23 (95% CI 21–26)	32.0	[13]
6.	Chile	622.391 South-Eastern area of Santiago	2012–2013	88	Clinical diagnosis, response to neostigmine, pyridostigmine or edrophonium test; electrophysiological studies, thorax CT scans, serological studies.	Facultad de Medicina, Departamento de Ciencias Neurológicas, Universidad de Chile. Servicio de Neurología, Hospital del Salvador. Clínica Las Condes, Santiago, Chile. The pyridostigmine Register.	-	8.39 (CI 95%, 7.98–8.80)	[14]
7.	China	13.000.000 - Guangzhou, Southern China	2000–2017	3707	Clinical features of MG, positive response to acetyl-cholinesterase inhibitors; decrements of 10%, increased jitter, positive anti-AChR antibody assay.	20 hospitals in Guangzhou.	0.15–0.36	2.19–11.07	[15]
8.	Faroe Islands	Nationwide	1986–2013	12	Clinical assessment, pharmacological test, anti-AChR antibodies.	The National Hospital of the Faroe Islands.	9.4 (95% CI 4.9–16.5).	18.7 (95% CI 8.3–35.1)	[7]
9.	Georgia	Nationwide	2008	161	-	All hospital cases recorded during 1954 - 2008.	-	3.1	[16]
10.	Hungary	Nationwide	2007	228/1439	International Classification of Diseases (ICD-10), code for MG.	The Hungarian Health Insurance Fund, Hospital Discharge Records.	2.76 (95% CI 2.40–3.12)	17.42 (95% CI 16.52–18.32)	[17]
11.	Republic of Ireland	Nationwide	2013	520	Demographic, clinical, electrophysiologic, histopathology, serology, and genetic data.	2 neuromuscular clinics in Beaumont Hospital, Dublin, and Cork University Hospital; referrals from neurologists and neurophysiologists; hospital databases; the voluntary patient organization Muscular Dystrophy Ireland (MDI); and the Hospital-In-Patient-Enquiry database (HIPE).	-	15.12 (95% CI 13.82–16.42)	[18]
12.	Israel	Nationwide	2004–2013	1842	Anti-AChR antibodies detected by radioimmunoassay.	Neuroimmunology laboratory at Hadassah Medical Center in Jerusalem. Har Hotzvim Laboratory in Jerusalem. The Clalit Laboratory of Neuroimmunology at the Rabin Medical Center. The laboratory of Meuhedet Medical Insurance.	18.49 (P<.0001)	-	[19]
13.	Italy	493.753 Pavia	2008	119	Osserman diagnostic criteria for primary autoimmune disease.	MG databases of the Neurological Institute of Pavia, MG Center of the ‘C. Mondino’ Neurological Institute of Pavia; General hospitals, neurological departments and clinics, rehabilitation institutions in the rest of Lombardy. Registries of the local health unit (list of anticholinesterase drug prescriptions, hospital discharge records, and mortality register).	-	24	[20]
14.	Italy	25.885	2009	-	MG patients registered in the “Millewin” software; clinical diagnosis according to National Programs on Clinical Guidelines.	Italian National Health System, data from 21 general practitioners.	-	35 (95% CI 16–66)	[21]
15.	Italy	524.826 [Statistic Center of Trento province in the 2009 census]	2005–2009	38	Clinical assessment, pharmacological tests, electrophysiological studies (repetitive nerve stimulation or jitter).	Hospital discharge diagnosis Information system. The database of the neuromuscular outpatient clinic of Trento Hospital. The database of the Immunology Laboratory of the University of Verona. Pharmacological Information System.	14.8 (95% CI 10.5–20.3)	12.9 (95% CI 10–16.4).	[22]
16.	Japan	Nationwide	2006	15.100	Clinical assessment, pharmacological test, electrophysiological studies, anti- AChR.	Research Committees of Neuroimmunological Diseases and of Epidemiology of Intractable Diseases.	-	11.8 (95% CI: 10.9–12.7)	[23]
17.	Korea	Nationwide	2011	1.236/5.410	Clinical records with a principal diagnosis of MG, the prescription of acetylcholinesterase inhibitors or immunosuppressive agents including corticosteroids and azathioprine within 2 years after the diagnosis.	NHI system, The Korean Health Insurance Review and Assessment Service.	0.24	10.66	[24]
18.	Latvia	Nationwide	2010–2014	99	Clinical assessment, pharmacological test, electrophysiological studies (repetitive nerve stimulation).	Neuromuscular Disease Clinic of Pauls Stradins Clinical University Hospital and Children’s Clinical University Hospital.	9.7 (95% CI 7.9–11.8)	11.3 (95% CI 9.9–12.9)	[25]
19.	Northern Europe (Norway vs. The Netherlands)	Nationwide	2010–2012	534 671	Clinical MG and presence of anti-AchR or -MuSK antibodies or >10% decrement on RNS or increased jitter on SF-EMG.	Department of Neurology, Oslo University Hospital, Department of Neurology, Leiden University Medical Centre, The Netherlands.	-	Norway: 13.8 (95% CI 12.6–15.0) The Netherlands: 16.7 (95% CI 15.5–18.0)	[26]
20.	Norway	Nationwide	2007	74	Prescriptions of pyridostigmine from a neurologist or prescription for MG as specified Norwegian Prescription Database (NorPD).	NorPD.	16.0	13.1	[27]
21.	Portugal	3.644.195 Northern Portugal	2013	23/407	Clinical records and pyridostigmine prescription registers.	Hospitals of the Regional Health Administration of the North Region. Private clinics. Computerized database of pyridostigmine prescriptions.	6.3	11.1	[28]
22.	Russian Federation	3 170 141, Samara	2000–2009	236	Clinical assessment, pharmacological tests, electrophysiological studies (repetitive nerve stimulation).	MG patients registry of the Samara region.	7.3	9.7	[29]
23.	Slovakia	Nationwide	1977–2015	2.074	Clinical diagnosis and at least 2 of the following criteria: positive antibodies to AChR or muscle-specific kinase, positive electrophysiological tests, positive reaction to anticholinesterase inhibitors.	Slovak Centre for Neuromuscular Diseases at the University Hospital Bratislava.	0.17	24.75	[30]
24.	South Africa	Nationwide	2011–2012	890	Anti-AChR antibodies.	National Health Laboratory Service. Laboratory of Drs. du Buisson, Kramer, Swart, & Bouwer, Inc.	8.5 ( 95% CI 8–9.1)	-	[31]
25.	Spain	155.069 County of Osona (Catalonia)	2013	51	Clinical diagnosis confirmed by the presence of acetylcholine receptor (AChR) or muscle-specific receptor tyrosine kinase (MuSK) antibodies in serum or Tensilon test and neurophysiological studies.	MG register, Neurology Department at Hospital General de Vic.	28.0	32.89 (95% CI 23.86–41.91).	[32]
26.	Sweden	Nationwide	2005–2010	2054	Diagnoses coded according to the Swedish revisions of the International Classification of Diseases, data on prescriptions of pyridostigmine and ambenonium.	Swedish health and population registers.	-	24.8	[33]
27.	Sweden	Nationwide	2006–2016	4736	Patients with classification codes for MG; and/or two or more prescriptions of pyridostigmine or ambenonium.	Swedish Health Registers.	29 (95% CI: 25–32)	36.1 (95% CI: 34.9–37.3).	[34]
28.	Taiwan	Nationwide	2000–2007	5.211	Cases of MG identified from the database corresponding to the ICD-9 codes.	National Health Insurance Research Database.	21.0	14.0	[35]
29.	Trinidad and Tobago	412.810 South Trinidad	2007–2010	36	Clinical diagnosis, serological tests, electrophysiological studies.	San Fernando General Hospital, SFGH outpatient neurology clinic and one private neurology practice in South Trinidad.	-	7.8	[36]

AChR – anti-acetylcholine receptor; CT – computed tomography; MRI – magnetic resonance imaging; MG – myastenia gravis; MuSK – muscle-specific kinase; RNS – repetitive nerve stimulation; SF-EMG – single fiber electromyography.

Data on MG incidence have varied over time and among different geographical regions, questioning whether there is a real geographical variation (that could point to the disease's etiology) or if this is due to methodological biases. Most epidemiological studies regarding rare and heterogeneous diseases such as MG bear limitations like small study populations, different inclusion criteria and sources of data, disparate diagnosis criteria, and often provide data that cannot be compared. Nationwide databases, including whole populations, offer a reliable ground for epidemiological studies but are not available in most countries.

The discrepancy in incidence due to methodological biases is expected to disappear as studies expand their year span and their quality enhances, thus possibly revealing real geographical trends.

Depending on the geographic location, the prevalence of MG ranges between 1.5 to 17.9 [1], or between and 2.19 to 36.71 cases/100.000 population (Table 1). This indicates an estimate of 56,000–123,000 patients in Europe [8] and 60.000 in the United States [5].

No epidemiological study has been performed in the Republic of Moldova so far; the only data available come from the neuromuscular division of the Institute of Neurology and Neurosurgery in Chisinau, where 320 patients are being supervised through an MG database, which includes patients diagnosed since 2008. One study determining the prevalence of multiple sclerosis in the Republic of Moldova has been performed [37], laying the ground for further similar epidemiological studies.

Epidemiological studies of MG in Kazakhstan have not been published yet. According to preliminary data, the prevalence ratio of MG in Kazakhstan is estimated to range from 0.5 to 5.0 per 100.000 people [38]. MG patients are being supervised through the Electronic Register of Dispensary Patients, which included 1187 registered patients in July 2019. In Almaty, the number of patients aged 18 years and older increased by 24% between 2017 and 2019. The largest number of patients was registered in Almaty (112 people), followed by the East Kazakhstan region - (103 patients) [38]. The growing number of cases and lack of comprehensive information on MG in Kazakhstan prompt to study it further to raise the effectiveness of diagnosis and treatment of this rare pathology and assess its social and economic implications.

Before 1934, the prevalence of MG was estimated at 1 in 200.000. After 1934, with the introduction of anticholinesterase compounds, it rose to 1 per 20.000 and 1 per 17.000 population after detecting serum antibodies against AChR in 1969 [39].

In the last decades, a further steady increase in prevalence has been observed [27]. The evidence comes from several geographic areas and diverse ethnicities [40]. Prolonged survival, a normal life expectancy, an aging population as well as improved diagnosis are probably the main explanations [9, 24]. Clinicians may be noticing that the natural history of the disease is different in older patients, questioning again whether MG is an umbrella term for several distinct diseases [5].

Currently, mortality from the disease is 5–9% [39], the overall in-hospital mortality rate is 2.2% and 4.7% in a myasthenic crisis. The most important predictors of death are age and respiratory failure [41]. The mortality rate is slightly higher in males (14%) than females (11%) [39]. The underlying or contributing cause of death among patients with MG are respiratory tract diseases, such as pneumonia and influenza. The occurrence of cardiac, cerebrovascular, and malignant diseases seems to be lower in MG patients [42]. Compared to 1934, when the few patients diagnosed with MG were those with extreme weakness and 70% of them died of respiratory failure or pneumonia within two years [39], the term “gravis” is now considered obsolete.

Overall, we expect to see more MG patients in the future [40]. This tendency implies that the future MG patient will be older and present with more comorbidities, which will lead to a more elaborate treatment.

### Ethnicity

A certain racial or ethnic background may influence MG’s presentation and course. For example, the age of onset is higher in Caucasians than non-Caucasians [43]. Also, the female gender is more prevalent among Hispanic, Asian, and African-American ethnicities compared to Caucasians [43].

The incidence rate of MG was reported to be higher in African-American women (0.01 per 1.000 population/year) compared to Caucasian women and Caucasian and African-American men (0.007-0.009 per 1.000 population/year) [41], probably because of a predisposition in the Black population for developing autoimmune diseases [43]. However, this study only dealt with hospitalized MG patients. There is also a higher incidence of ocular MG among Black men and women [44], while Caucasian women show higher rates of generalized MG [43].

A multi-racial study has been conducted in South Africa [45], showing that Black subjects of indigenous African origin were more likely than Whites to develop ocular MG. White subjects were more likely than Black subjects to develop severe generalized MG that was poorly responsive to treatment and repeated myasthenic crises [45].

Asian populations report more cases of early-onset MG, which is more often ocular MG [3]. Juvenile-onset and particularly infantile-onset ocular myasthenia (0–4 years) [23] is also more common in Asian populations, with up to 30% of patients manifesting this form [3]. This epidemiological particularity has been associated with HLA-Bw46 and DR9 [3]. Only 50% of juvenile-onset MG in Asian populations are anti-AchR positive [46].

In a Japanese epidemiological study [23], 80.6% of children in the infantile-onset group presented with the ocular form, compared to 14–30% in Europe and North America. A similar high frequency of juvenile-onset ocular MG was reported in Chinese populations, suggesting a distinct immunological mechanism [23].

The late-onset peak of MG is less expressed in Japanese, Indian and Chinese patients with MG [3]. A high proportion of patients from Asia presents higher rates of MuSK MG compared with Caucasian MG patients [47].

Ethnic epidemiological particularities were also studied in a cohort of Jewish Ashkenazi (ASH) and non-Ashkenazi (NASH) because of their different origins [47]. ASH Jews originated in Europe, while NASH Jews originated in the Middle East and North Africa, having a more diverse ancestry. The results revealed a higher frequency of ocular MG in the ASH group [47].

Studies of migrating populations are useful for exploring the genetic and environmental aspects of multifactorial diseases. A study performed in Norway and Netherlands tried to determine the epidemiological characteristics of MG in immigrants compared to native patients [26]. The study showed that MuSK MG and MG with thymoma were more frequent in Asian immigrants in comparison to other racial types indicating that these patients bring genetic elements or lifestyle particularities acquired before the migration that support their distinctive phenotype.

### Age and gender

MG affects all ages, but it is considered “a disease of young women and old men”. The earliest age of onset reported is 1 year [48], while the oldest onset age reported was 98 years old [3]. There has been a constant increase of late-onset MG in Western [23] and Asian [49] countries over the last 30 years. The incidence of MG patients with an onset age of 50 years or more increased by 1.5-fold, while the number of patients with an onset age of 65 years or more showed a 2.3-fold rise [23]. This increase is probably due to better recognition of the disease and improved diagnostic tests. However, it may also suggest a distinct immunological background in the elderly or that environmental factors may be involved.

The most common onset age is between 20 and 39 years in women [50] and between 50 and 70 years in men [3]. The onset age curve is constantly bimodal for females, with an early-onset and a late-onset peak, and tends to present only one late-onset peak for males. The cause of this distribution has haunted researchers for many years. Gender is a deciding epidemiological risk factor for the development of autoimmune diseases, given that the immune system is a subject of sexual dimorphism [51]. Many autoimmune diseases, including MG, are more prevalent in women than in men. In early-onset MG, the female:male ratio is 3:1 [52]. These observations raise the question of estrogens as mediators of sex differences in autoimmunity [53].

Acquired MG may manifest for the first time during pregnancy [3]. Pregnancy has a variable effect on the disease; 41% of patients will present exacerbation, 29% will show remission, and no change will be present in the course of the disease of 30% [6]. Also, women often describe a worsening of the symptoms before their menstrual period, when progesterone levels are lowering [54].

Thymic hyperplasia affects female patients (ratio 9:1) during the fertile period of their life [55]. Estrogen receptors are expressed on thymic epithelial cells and thymocytes [52]. An increased expression of estrogen receptors was found on the thymocytes and T cells from peripheral blood mononuclear cells in MG patients [55]. Potentially, the B cells of MG patients show an aberrant reactivity to estrogens and an intensified B-cell response [55].

Androgens suppress both T-cell and B-cell immune responses, resulting in the suppression of disease expression serving as a protective factor [51]. The unimodal distribution among males might be explained by an age-related decline in testosterone [56]; however, this may also be coincidental.

### Risk factors: genetic architecture, geo-epidemiology and the environment

MG is very rarely inherited, between 3.8% and 7.1% of MG patients reporting a family history of the disease [57]. Few cases of familial autoimmune MG have been reported: a family with parental consanguinity and five of 10 siblings affected by late-onset autoimmune MG [58], a Hungarian family where nine members from two generations developed MG [6], an Italian-American family with 5 children affected by early-onset MG linked to a variant in the ecto-NADH oxidase 1 gene (ENOX1) [6]. Monozygotic MG twin concordance is estimated at 35%, supporting a genetic influence in disease development [57].

Even though MG is rarely inherited, other autoimmune diseases, such as thyroid disorders and rheumatoid arthritis, are frequent among the MG patients and the myasthenic patient’s relatives, suggesting that both genetics and the environment are predisposing factors [6]. Autoimmune thyroiditis occurs in 10% of MG patients [59].

Genetic susceptibility in patients with MG has been suggested in different human leukocyte antigens (HLA) associations. The correlation with HLA fluctuates widely in relation to sex, age at disease onset, and thymic histology of the patients, and it differs between Caucasian and Asian populations [60]. One important and consistent feature in MG is the association with different HLA antigens in different ethnic groups.

The HLA A1-B8-DR3-DQ2 haplotype, also known as AH8.1, is associated with early-onset MG in the Caucasian population [6], while late-onset MG is associated with a variety of other HLA markers [3]. Patients with MuSK-MG seem to be associated with HLA-DR and DQ5 alleles [6, 42]. AH8.1 has also been associated with systemic lupus erythematosus (SLE) and celiac disease, suggesting a shared genetic background for autoimmune diseases [6]. The DQ9 and HLA-DRB1(*)09 haplotypes were associated with MG in Asian patients [6]. Although MG patients with diversified ethnic backgrounds carried different HLA haplotypes, several loci (HLA-B*08 and HLA-DQA1) presented virtually universally in certain MG subtypes. The similarities in HLA profiles conform to global human migration trajectories, during which the people with the same ethnic background carried common ancestral haplotypes [56].

Early-onset MG, which occurs more frequently among young women, is associated with HLA-A1, B8, -DR3 alleles, whereas late-onset MG occurs predominantly in men who demonstrate associations with HLA- A3, B7, -DR2 alleles [8]. Some alleles are suggested to be protective for men with MG [61].

In an individual with a susceptible genotype, exposure to environmental factors can act to initiate an autoimmune process [62]. The gene variants that make an individual susceptible or protect him from autoimmune disease development are probably a result of evolutionary adaptation to the environment [8]. Environmental factors, such as stressful life events, viral infections, and various drugs or toxins, have been postulated to precipitate the development of MG. Some patients report that the disease is precipitated by a viral or bacterial respiratory or other infection (4%), emotional stress (4%), a physical trauma (3%), hyperthyroidism (2%), or administration of thyroid hormones (1%), operative procedures especially thyroidectomy (1%), pregnancy or delivery (1%), allergic reactions (1%), or exposure to drugs such as quinidine, procainamide, penicillamine, aminoglycoside, or other antibiotics (1%) [39].

A correlation between autoimmune diseases and infections has been suggested, including hepatitis C, herpes simplex virus, Epstein-Barr virus, Cytomegalovirus, Human T-lymphotropic virus, West Nile Virus [46], and more recently SARS-CoV-2 [63]. SARS-CoV-2 may exacerbate MG [64] and also trigger it. Three cases of AChR antibody-positive MG associated with SARS-CoV-2 were described in August 2020 [63], with MG symptoms occurring within 5–7 days after the onset of fever in patients without previous neurological or autoimmune disorders. The “hygiene hypothesis” suggests that a better quality of life and a fewer incidence of infections like tuberculosis in the developed world explain the rise in incidence and prevalence of MG. However, data from countries with high rates of infectious diseases and similar MG trends do not uphold this theory [8].

A multitude of toxins from animal, vegetal, and bacterial sources are able to disturb the function of the NMJ. Insecticides used in agriculture act on nerve targets, such as acetylcholinesterase, pseudocholinesterase, and the AChR [65]. This leads to questioning whether recurrent exposure to pesticides could cause MG (by sensitizing the immune system to the cholinergic receptor) [65]. In this regard, several studies have been conducted in agricultural populations. One of them revealed an increased prevalence of MG in adult men from rural areas in Morocco, including workers exposed to pesticides suspected of triggering autoimmune diseases, suggesting a role of these substances [66]. Another study detected an increased prevalence of MG among rural male adults in Israel, associating it with pesticide exposure [65]. It cannot be ruled out that other factors may explain this association, but physicians might consider pesticide exposure when patients present with MG symptoms [65].

## Conclusions

Epidemiological data regarding MG are important for postulating hypotheses regarding its etiology and facilitating the description of MG subtypes. Thus, adequate documentation through broad databases is essential.

The incidence and prevalence of MG reported around the globe have been rising steadily and consistently over the past decades. We expect the future MG patient to be older and have more comorbidities, which will lead to a more elaborate treatment.

Ethnic differences have been described, suggesting that genetic or environmental/lifestyle factors contribute to a specific phenotype, but also that MG may be considered as a complex of symptoms associated with different genetic susceptibility rather than a single clinical entity.

MG is still a disease of young women and older men. Gender is a deciding epidemiological risk factor for the development of autoimmune diseases, given that the immune system is a subject of sexual dimorphism. Many environmental factors are associated with MG, supporting the hypothesis that genetically sensitive individuals develop the autoimmune disease after exposure to environmental triggers.

## Acknowledgments

### Conflict of interest

The authors declare that there is no conflict of interest.
